# Pyrexia: aetiology in the ICU

**DOI:** 10.1186/s13054-016-1406-2

**Published:** 2016-09-01

**Authors:** Daniel J. Niven, Kevin B. Laupland

**Affiliations:** 1 Department of Critical Care Medicine and Community Health Sciences, O’Brien Institute for Public Health, Cumming School of Medicine, University of Calgary, Calgary, AB Canada; 2 ICU Administration, Foothills Medical Centre, 3134 Hospital Drive NW, Calgary, AB T2N 2T9 Canada; 3 Department of Medicine, Royal Inland Hospital, 311 Columbia Street, Kamloops, BC V2C 2T1 Canada

**Keywords:** Pyrexia, Fever, Hyperthermia, Temperature, ICU, Etiology, Aetiology, Cause, Incidence

## Abstract

Elevation in core body temperature is one of the most frequently detected abnormal signs in patients admitted to adult ICUs, and is associated with increased mortality in select populations of critically ill patients. The definition of an elevated body temperature varies considerably by population and thermometer, and is commonly defined by a temperature of 38.0 °C or greater. Terms such as hyperthermia, pyrexia, and fever are often used interchangeably. However, strictly speaking hyperthermia refers to the elevation in body temperature that occurs without an increase in the hypothalamic set point, such as in response to specific environmental (e.g., heat stroke), pharmacologic (e.g., neuroleptic malignant syndrome), or endocrine (e.g., thyrotoxicosis) stimuli. On the other hand, pyrexia and fever refer to the classical increase in body temperature that occurs in response to a vast list of infectious and noninfectious aetiologies in association with an increase in the hypothalamic set point. In this review, we examine the contemporary literature investigating the incidence and aetiology of pyrexia and hyperthermia among medical and surgical patients admitted to adult ICUs with or without an acute neurological condition. A temperature greater than 41.0 °C, although occasionally observed among patients with infectious or noninfectious pyrexia, is more commonly observed in patients with hyperthermia. Most episodes of pyrexia are due to infections, but incidence estimates of infectious and noninfectious aetiologies are limited by studies with small sample size and inconsistent reporting of noninfectious aetiologies. Pyrexia commonly triggers a full septic work-up, but on its own is a poor predictor of culture-positivity. In order to improve culturing practices, and better guide the diagnostic approach to critically ill patients with pyrexia, additional research is required to provide more robust estimates of the incidence of infectious and noninfectious aetiologies, and their relationship to other clinical features (e.g., leukocytosis). In the meantime, using existing literature, we propose an approach to identifying the aetiology of pyrexia in critically ill adults.

## Background

Temperature is commonly measured as part of the routine assessment of patients admitted to adult ICUs. Normal body temperature is between 36.0 and 37.5 °C [1]. Elevated body temperature is detected in approximately 50 % of patients admitted to adult ICUs [2–6]. In addition to being common, elevated body temperature is associated with increased mortality in subpopulations of critically ill patients including those with acute neurological conditions [7–9], noninfectious aetiologies of pyrexia [10], and medical patients that develop pyrexia in the ICU [3]. Although the optimal approach to managing elevated body temperature in critically ill patients remains controversial [11–15], it is widely accepted that elevated body temperature is an evolutionarily conserved sign of an underlying physiologic stressor and its presence should trigger a systematic search for the aetiology.

In this article, we will review the contemporary literature investigating the aetiology and incidence of elevated body temperature among patients admitted to adult ICUs. We will begin by examining literature pertaining to the measurement of body temperature and definitions for what constitutes an elevated temperature. We will then focus on the aetiology of pyrexia in medical and surgical patients with and without acute neurological conditions, including a brief discussion on hyperthermia syndromes. Because immunocompromised patients present a distinct set of aetiologic considerations, they will not be reviewed.

## Literature review

Relevant articles were identified through three sources. First, a semistructured literature review was conducted using MEDLINE from 1966 to 15 January 2016. The objective of the search was to identify English-language articles that reported on the aetiology or incidence of pyrexia among medical and surgical patients admitted to adult ICUs. The search used a combination of exploded Medical Subject Heading terms and text words that included synonyms for pyrexia and ICU, and a validated search filter for identifying studies investigating aetiology [16]. Titles and abstracts were screened for relevance, and appropriate full-length articles were retrieved for appraisal. Second, additional articles were identified using the “related articles” feature in PubMed and by hand-searching bibliographies of included studies, previously published reviews [17, 18], and relevant societal guidelines [19]. Finally, the authors’ personal files were screened for other articles of relevance.

## Body temperature measurement and definitions

**Fig. 1:**
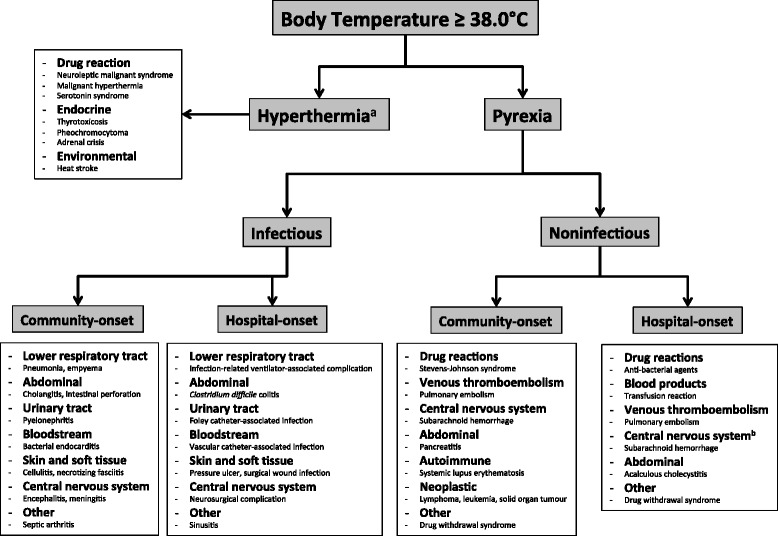
Approach to determining the aetiology of elevated body temperature in immunocompetent patients admitted to adult ICUs. Example diagnoses are included in each of the terminal boxes. ^a^Often associated with a temperature of 41.0 °C or greater. ^b^Onset will be later for a small percentage of patients with “central pyrexia”, otherwise this most commonly occurs early during ICU admission [45, 51]

Normal body temperature is between 36.0 and 37.5 °C, with intraindividual variability of 0.5–1.0 °C depending on the time of day (low in early morning, peak in early afternoon/late evening) [1, 20]. Elevated body temperature is classified as pyrexia or hyperthermia. Although these two terms are often used interchangeably, their biological mechanisms and response to therapy are different—thus their distinction is important and will be maintained in this article. Pyrexia, also referred to as fever, is an adaptive response to a physiologic stress that is tightly regulated through endogenous pyrogenic and anti-pyretic pathways, and is associated with an increase in the hypothalamic set point [18]. As such, the elevated body temperature in patients with pyrexia responds to pharmacologic anti-pyretic therapies such as acetaminophen. On the contrary, the elevated body temperature that occurs in hyperthermia syndromes often exceeds 41.0 °C, and reflects a pathologic increase in body temperature that is not associated with an increased hypothalamic set point [21]. This elevated temperature in hyperthermia is therefore not responsive to pharmacologic anti-pyretic therapy. For this article, pyrexia and fever will be used interchangeably, but hyperthermia will refer to the syndrome that accompanies specific environmental, pharmacologic, or endocrine stimuli (Fig. 1).

There is considerable heterogeneity among clinicians in the temperature threshold used to define an episode of pyrexia or hyperthermia [22]. This heterogeneity includes not only temperature variability related to the normal diurnal variability in body temperature, but also differences in the thermometer used to measure temperature. Guidelines in critically ill adults recommend measuring temperature using a central thermometer that provides a direct measure of the core temperature [19]. Examples of such central thermometers include the pulmonary artery catheter, and urinary bladder, esophageal, and rectal thermistors. Although these thermometers provide the most accurate assessment of core body temperature, they are not commonly employed as the primary method of temperature measurement in critically ill patients. Rather, temperature is commonly measured using thermometers that measure temperature from a peripheral site (e.g., tympanic membrane, temporal artery, axilla, mouth) and use proprietary algorithms to convert the measured temperature into a core temperature [22, 23]. Unfortunately, peripheral thermometers are among the least accurate, especially in patients with fever or hypothermia. A recent systematic review and meta-analysis found that, compared with central thermometers, the sensitivity and specificity of peripheral thermometers for the detection of fever was 64 % (95 % CI 55–72 %) and 96 % (95 % CI 93–97 %), respectively [23]. Therefore, for patients where exact temperature measurement is critical or when measured temperature is not congruent with the clinical picture, confirmation with a central device is a key step in determining the underlying aetiology.

A systematic review of observational studies in febrile critically ill adults reported five different definitions of pyrexia among the nine included studies, with 38.3 °C being the most frequently cited threshold [4]. A meta-analysis examining thermometer accuracy found seven distinct definitions of pyrexia, with 37.8 °C being the most frequently cited temperature threshold [23]. A multinational survey of 139 ICUs in 23 countries found 14 discreet temperature thresholds used to define pyrexia with a range of 37–40 °C and a median (interquartile range) of 38.2 °C (38.0–38.5 °C) [22]. Guidelines in critically ill adults define pyrexia as a temperature of 38.3 °C or greater, with the caveat that a lower threshold should be used in immunocompromised patients who are more likely to harbor a severe illness without significant elevation in body temperature [19]. Although not a well-established inherited or acquired state of immunodeficiency, being critically ill presents multiple factors that may impair immune system function and the likelihood of responding to physiologic stress through an elevation in body temperature. These factors include but are not limited to the presence of invasive catheters, decreased mobility and ability to communicate, frequent use of anti-pyretic analgesic drugs and/or broad-spectrum antimicrobials, and extracorporeal forms of organ system support. Therefore, in this article a core body temperature of 38.0 °C or greater will represent pyrexia or hyperthermia. High pyrexia will be defined as a temperature of 39.5 °C or greater [18].

## Aetiology and incidence of hyperthermia and pyrexia

The absolute body temperature and the response to pharmacological anti-pyretic therapy are often useful in distinguishing between hyperthermia and pyrexia. A temperature that exceeds 41.0 °C and is not responsive to pharmacologic anti-pyretic therapy is more commonly observed in patients with hyperthermia. Therefore, patients with milder elevations in body temperature and/or those whose temperature decreases when administered pharmacologic anti-pyretic drugs are unlikely to be suffering from a hyperthermia syndrome. On the contrary, the absolute temperature is not as helpful in differentiating between infectious and noninfectious pyrexia. Rather, this requires careful examination and investigation for a broad number of infectious and noninfectious diagnoses (Fig. 1). The following sections will provide an overview of several common aetiologies of hyperthermia and pyrexia, followed by presentation of data supporting their incidence in medical, surgical, and neurologically impaired patients.

### Hyperthermia syndromes

The hyperthermia syndromes, generally characterized by severely elevated body temperature, include: environmental hyperthermia including heatstroke [24]; drug-induced hyperthermia, including malignant hyperthermia [21], neuroleptic malignant syndrome [25], and serotonin syndrome [26]; and endocrine causes including thyrotoxicosis, pheochromocytoma, and adrenal crisis [27]. Heatstroke is defined clinically as a core temperature greater than 40 °C associated with central nervous system impairment and multisystem organ failure [24]. Classic heat stroke occurs among older, chronically ill, and debilitated individuals during heat waves, wherein a high external temperature overwhelms the body’s thermoregulatory capacity to dissipate the heat [28]. Exertional heat stroke typically occurs in young, otherwise healthy individuals undergoing strenuous physical activity, wherein the excessive heat production disrupts usual thermal homeostasis [28]. Complications of heat stroke include rhabdomyolysis, disseminated intravascular coagulation, renal and liver failure, and severe metabolic derangements including hypoglycemia, lactic acidosis, and hyperkalemia [24]. Malignant hyperthermia occurs among patients with inherited mutations in the ryanodine receptor and is characterized by the acute onset of muscle rigidity, hyperthermia, and acidosis in response to exposure to inhalational anesthetics or depolarizing paralytic medications [21]. Neuroleptic malignant syndrome is characterized by the insidious onset of muscle rigidity, hyperthermia, and mental status changes that occur because of the administration of centrally-acting dopamine antagonists, usually typical or atypical antipsychotics, but may also include anti-nauseant medications such as metoclopramide [25]. Serotonin syndrome presents with the rapid onset of hyperthermia and other signs of autonomic instability including tachycardia, mydriasis, and diaphoresis, as well as cognitive and neuromuscular changes that may include tremor, hyperreflexia, and clonus in patients with excess central and peripheral serotonergic agonism [26]. Typically, serotonin syndrome occurs following intentional self-poisoning with prescription or illicit serotonergic agents, but may also occur in the context of therapeutic drug use, including antibiotic therapy with linezolid, or inadvertent drug interactions [26]. Endocrine emergencies occasionally present with hyperthermia. While less commonly the case for pheochromocytoma and adrenal crisis, hyperthermia is a common feature of severe thyrotoxicosis, and is one of the diagnostic features of thyroid storm [27].

### Pyrexia due to infectious aetiologies

Many episodes of pyrexia are due to infections, and can be broadly divided according to whether they are community onset or hospital onset, with hospital-onset infections manifesting 48 hours or more after admission to hospital [29]. Whether community onset or hospital onset, the most common source of infection in immunocompetent critically ill patients is the lower respiratory tract [12, 30]. For patients admitted from the community this typically represents an infectious bronchitis, or community-acquired pneumonia with or without its associated complications such as abscess or empyema. Most lower respiratory tract infections that take origin in the community are due to a viral pathogen such as human rhinovirus or influenza A/B, with a smaller portion being bacterial, such as *Streptococcus pneumoniae*, less commonly mycobacterial, or rarely fungal [31]. For mechanically ventilated patients, hospital-onset lower respiratory tract infection is typically an infectious tracheobronchitis or ventilator-associated pneumonia. These events are usually due to bacteria such as *Staphylococcus aureus*, Gram-negative bacilli, and less frequently fungal aetiologies.

Peritonitis due to intra-abdominal infection is a common community-onset infectious syndrome [30]. Frequently encountered diagnoses include intestinal perforation, intestinal ischemia with consequential perforation or secondary bacterial invasion of the bloodstream, cholecystitis, appendicitis, perforated diverticular abscess, or hepatic abscess. For patients recovering from abdominal surgery, especially those with intestinal perforation who may have had contamination of the peritoneal space with intestinal contents, intra-abdominal abscess should be sought as a source of pyrexia; otherwise this is generally an uncommon source of new hospital-onset infection. However, colitis due to *Clostridium difficile* infection should not be overlooked in the hospitalized patient with fever and diarrhea, especially in patients who have been treated with broad-spectrum antibiotics [32].

Urinary tract infection is another common aetiology of pyrexia [12]. While the majority of community-onset lower urinary tract infections are not associated with fever and systemic symptoms, patients admitted to hospital and especially the ICU owing to a urinary tract infection typically have infection of the upper urinary tract wherein high fever and other signs of sepsis are common. In patients whose urinary tract infection develops while in the ICU, this is usually the result of an accumulated biofilm on the urinary bladder catheter. In these patients, not only is the bladder catheter the source of the infection, it may also mask the development of symptoms classically ascribed to infection of the urinary tract, and pyrexia may be the only presenting sign [33]. For community-onset infections, the organisms most frequently detected include *Escherichia coli*, *Klebsiella pneumoniae*, and *Enterococcus* species [34], whereas *Pseudomonas aeruginosa* is frequently isolated among patients with a urinary tract infection acquired in the ICU [35].

Any infection, but especially those in the lower respiratory tract, abdomen, or urinary tract, can invade the bloodstream. Given that bloodstream infection can also be primary or catheter related, the bloodstream is a common site of infection in critically ill patients with pyrexia [3, 5, 19, 36]. Among patients admitted to the ICU owing to a severe bloodstream infection that is community onset, the most common pathogens are *E. coli*, *S. aureus*, and *S. pneumoniae* [37]. For those infections that are hospital onset, *S. aureus* and *E. coli* are also the two most common pathogens, followed by Gram-negative Enterobacteriaceae and *Enterococcus faecalis* [37]. Risk factors for developing a severe bloodstream infection (community or hospital onset) include older age and the presence of underlying medical comorbidities especially diabetes mellitus, dialysis-dependent renal failure, cancer, lung disease, and alcoholism [37]. Fungemia with *Candida albicans* and non-albicans *Candida* species is important to consider as a cause of pyrexia that develops in hospital, especially in patients with risk factors such as recent surgical operation, sepsis, treatment with parenteral nutrition, and/or broad-spectrum antibacterial agents. Although rates of catheter-related bloodstream infection have decreased considerably owing to use of infection prevention bundles and early removal of unnecessary catheters [38, 39], this remains an important source of pyrexia in patients admitted to ICUs. While classically thought to present negligible risk, the infectious risk associated with arterial catheters is similar to central venous catheters [40, 41]. Coagulase-negative staphylococci are the most common pathogen isolated in patients with catheter-related bloodstream infection, with other common organisms including *S. aureus*, enterococcal species, and Gram-negative Enterobacteriaceae [42, 43].

Other sources of infection include the skin and soft tissue, bone/joints, central nervous system, and ethmoid and maxillary sinuses [30]. Diagnoses such as cellulitis and necrotizing fasciitis commonly take origin in the community, although they may rarely be hospital onset. Skin breakdown particularly in the sacral area is a common problem in patients with long ICU or hospital lengths of stay, and these areas may become infected. In addition, any operative wound can become colonized and ultimately infected. Hospital-onset infections of the central nervous system are uncommon outside the neurosurgical setting; however, among patients with persistent bacteremia and prolonged pyrexia, consideration should be given to potential seeding of the paraspinal and/or epidural spaces. Finally, for patients with prolonged insertion of nasogastric and/or nasotracheal tubes, sinusitis commonly develops, and may be responsible for pyrexia that is not accompanied by other systemic signs of infection [44].

### Pyrexia due to noninfectious aetiologies

Noninfectious diagnoses are also common causes of pyrexia in adult ICUs, especially among patients with an acute neurological condition [45]. Unless there are obvious signs and symptoms of the particular noninfectious problem, such as an exanthem in the context of a drug reaction or asymmetric leg edema in the context of deep vein thrombosis, these diagnoses are often not made until infectious aetiologies have been ruled out by detailed examination and an appropriate set of investigations. Similar to infectious aetiologies, noninfectious aetiologies can be grouped according to community or hospital onset. Although there may be a fair bit of overlap between the types of noninfectious community and hospital-onset problems responsible for pyrexia, this distinction is important because autoimmune and neoplastic aetiologies rarely develop in hospital. On the contrary, blood products and medications are commonly prescribed to hospitalized patients, and thus transfusion reactions and drug hypersensitivity, in particular to antimicrobial or antiepileptic agents, are frequent causes of noninfectious fever that develops in hospital. In addition, despite meticulous attention to appropriate prescription of venous thromboembolism prophylaxis, deep vein thrombosis and pulmonary embolism can be the source of a new episode of pyrexia, especially if complicated by septic thrombophlebitis. Abdominal sources may also be responsible for a noninfectious fever. Patients admitted to the ICU with pancreatitis may be febrile at ICU admission; however, fever may recur in hospital if pancreatitis is severe and complicated by necrosis and/or pseudocyst formation. While acalculous cholecystitis is not a typical diagnosis at ICU admission, it commonly develops in patients recovering from nonbiliary surgery and/or in those with significant periods of hypotension [46]. Although the mechanism behind the development of early postoperative fever remains unclear, and may or may not involve lung atelectasis [47], early-onset fever is commonly noninfectious in origin for patients admitted to the ICU following elective surgery [2]. For patients admitted to the ICU with a neurological condition such as subarachnoid hemorrhage, traumatic brain injury, or intracerebral hemorrhage, pyrexia that occurs within the first couple days of ICU admission is most likely central fever due to the temperature dysregulation associated with the neurological injury rather than another infectious or noninfectious process.

### Incidence of pyrexia and aetiologies

The incidence of pyrexia among critically ill adults depends on the defining temperature threshold and the population studied (Table 1). A systematic review of nine observational studies in patients admitted to the ICU without an acute neurological condition found that the fever incidence varied between 26 and 88 % [4]. The largest studies were from Barie et al. [2] and Laupland et al. [3]. Defined as a temperature of 38.2 °C or greater, Barie and colleagues found that fever was present in 26 % of 2419 patients admitted to their surgical ICU over a 14-month period [2]. Infections were responsible for 46 % of febrile episodes, and were more likely in patients whose fever occurred at the time of admission following emergency surgery. On the contrary, among patients admitted to the ICU following elective surgery, early fever within 72 hours of admission was more likely to be noninfectious, and infectious aetiologies did not emerge until after 72 hours in the ICU. Defined as a temperature 38.3 °C or greater, Laupland and colleagues found that the cumulative incidence of fever was 44 % among 20,466 critically ill adults with a broad range of admission diagnoses, with the highest incidence among trauma/neurologic patients [3]. Cultures from blood, urine, sputum, cerebrospinal fluid, and/or other sterile fluid were positive in 17 % and 31 % of fever and high fever episodes, respectively [3]. Bloodstream infection occurred in 9 % and 19 % of fever and high fever episodes, respectively. Culture-positivity was most likely among medical patients. However, this study probably underestimated the incidence of infectious fever because the authors did not systematically evaluate the results of diagnostic tests other than cultures for identifying infection [3]. Another study in the same health region that undertook a detailed chart review to investigate pyrexia management practices in 100 medical and surgical critically ill patients without an acute neurological condition found that infections were responsible for 73 % of pyrexia episodes, with pneumonia the most common infection, occurring in 70 % of infectious fevers [36]. This is consistent with a large, prospective study of infection occurrence in 71 ICUs in Italy, wherein the most common source of infection was pneumonia [30].

**Table 1:** Studies reporting the aetiology of pyrexia in immunocompetent patients admitted to adult ICUs with or without an acute neurological condition

						Aetiology	
Study	Setting	Design	Total patients (*n*)	Episodes of pyrexia (*n*)	Pyrexia definition (°C)	Infectious diagnosis (*n*, %)^a^	Noninfectious diagnosis (*n*, %)^a^
No acute neurological condition							
Circiumaru et al., 1999 [56]	Medical–surgical ICU	Prospective observational study	93	70	≥38.4	Total (37, 53) Respiratory (15, 21) BSI (9, 13) Abdominal (5, 7) Other (8, 11)	Total (33, 47) ARDS (4, 6) MI (3, 4) Vasculitides (2, 3) Pancreatitis (1, 1) Atelectasis (1, 1) GVHD (1, 1) ICH (1, 1) Unclear (20, 29)^b^
Peres Bota et al., 2004 [57]	Medical–surgical ICU	Prospective observational study	493	139	≥38.3	Total (76, 55)	Total (63, 45) Postoperative (27, 19) Cerebral hemorrhage (20, 14) Trauma (5, 4) ARDS (3, 2) MI (2, 1) Pancreatitis (3, 2) GI bleed (3, 2)
Barie et al., 2004 [2]	Surgical ICU	Prospective observational study	2419	626	≥38.2	Total (286, 46)^c^	Total (330, 53)^c^
Laupland et al., 2008 [3]	3 medical–surgical ICUs, 1 CVICU	Retrospective observational study	20,466^d^	10,730	≥38.3	Culture-positive (1847, 17) BSI (1004, 9)	Culture-negative (8883, 83)
Niven et al., 2011 [36]	3 medical–surgical ICUs, 1 CVICU	Retrospective observational study	7535	100^e^	≥38.3	Total (73, 73) Pneumonia (51, 51) BSI (6, 6) Other (15, 15)	Total (27, 27)
Gozzoli et al., 2001 [58]	Surgical ICU	RCT	38	38	≥38.5	Total (18, 47)	Total (20, 53)
Niven et al., 2013 [59]	2 medical–surgical ICUs	RCT	26	26	≥38.3	Total (23, 88) Respiratory (15, 58) UTI (2, 8) BSI (1, 4) Other (5, 19)	Total (3, 12)
Schortgen et al., 2012 [11]	7 medical–surgical ICUs	RCT	200	200	>38.3	Total (200, 100)^f^ Lungs (138, 69) Abdomen (13, 7) Genitourinary (12, 6) Other (28, 14) Unknown (9, 5)	Not applicable
Young et al., 2015 [12]	23 medical–surgical ICUs	RCT	700	700	>38.0	Total (700, 100)^f^ Respiratory (237, 34) Abdominal (92, 13) UTI (68, 10) BSI (42, 6) Skin/soft tissue (54, 8) Other (207, 30)	Not applicable
Acute neurological condition							
Commichau et al., 2003 [49]	Neurological ICU	Prospective observational study	387	87	≥38.3	Total (45, 52)^g^ Respiratory (37, 42) *Clostridium difficile* (4, 4) UTI (3, 3) Sinusitis (1, 1)	Total (2, 2) DVT (2, 2)
Rabinstein et al., 2007 [45]	Neurological ICU	Prospective observational study	93	93	≥38.3	Total (62, 67) Respiratory (46, 49) Other (16, 17)	Total (31, 33) Central fever (27, 29) Alcohol withdrawal (3, 3) Phenytoin toxicity (1, 1)
Hocker et al., 2013 [51]	Neurological ICU	Retrospective observational study	526	526	>38.3	Total (280, 53)	Total (246, 47)

^a^Proportion refers to percentage of total number of pyrexia episodes

^b^Inconsistencies in reporting of pyrexia aetiologies; total number infectious and noninfectious aetiologies did not total the number of pyrexia episodes

^c^Detailed data for infectious and noninfectious aetiologies presented in graphical format only

^d^A total of 24,204 ICU admissions among 20,466 patients

^e^Convenience sample of 100 randomly selected patients. Total number of patients with fever during study period was 2216

^f^Both Schortgen et al. [11] and Young et al. [12] preferentially enrolled patients with suspected or confirmed infection

^g^Forty-two pyrexia episodes did not have a clear aetiology

*ARDS* acute respiratory distress syndrome, *BSI* bloodstream infection, *CVICU* cardiovascular intensive care unit, *DVT* deep vein thrombosis, *GI* gastrointestinal, *GVHD* graft versus host disease, *ICH* intracerebral hemorrhage, *MI* myocardial infarction, *RCT* randomized controlled trial, *UTI* urinary tract infection

Unfortunately, the current literature does not consistently document the incidence of noninfectious aetiologies of pyrexia. Similarly, the incidence of the hyperthermia syndromes among ICU patients with an elevated body temperature is not clear.

Among critically ill patients with an acute neurological condition, the reported incidence of fever varies between 23 and 51 % [6, 48–50]. The largest study investigating the epidemiology of fever among patients admitted to the ICU with an acute neurological condition is that of Rincon et al. [6]. Of 13,587 patients admitted to 94 ICUs in the United States, 6965 (51 %) had fever defined as a temperature of 37.5 °C or greater. The incidence of fever was highest among patients with traumatic brain injury (60 %) or aneurysmal subarachnoid hemorrhage (54 %), and lowest among patients with acute ischemic stroke (37 %) [6]. Unfortunately, Rincon and colleagues did not report on the aetiologies of pyrexia, and therefore estimates of the occurrence of infectious and noninfectious fever in neurological patients derive from smaller studies (Table 1). Among 93 patients admitted to a neurological ICU in the United States who developed pyrexia, infection was the cause in 62 patients (67 %) with noninfectious causes accounting for the other 31 (33 %) patients [45]. Infections were more common in patients with traumatic brain injury, whereas a noninfectious cause, most probably central fever, was more likely in patients with subarachnoid hemorrhage. Admission diagnosis of subarachnoid hemorrhage (odds ratio (OR) 11.79, 95 % CI 3.0–59.4) and fever onset within 72 hours of ICU admission (OR 2.21, 95 % CI 1.22–4.34) were statistically significant predictors of noninfectious fever [45]. Hocker and colleagues examined the occurrence of infectious and noninfectious aetiologies of fever in 526 patients admitted to a neurological ICU in the United States [51]. Because their objective was to develop a model predictive of the probability of central fever, they unfortunately excluded patients with other noninfectious causes of pyrexia. Nonetheless, they found that fever was infectious in 53 % and central in 47 %. The combination of negative cultures (urine, blood, respiratory secretions, cerebrospinal fluid, peritoneal fluid, stool, sinus aspirates, and *C. difficile* PCR), an absence of infiltrate on chest radiograph, the diagnosis of subarachnoid hemorrhage, intraventricular hemorrhage, or tumor, and the onset of fever within 72 hours of admission predicted central fever with a probability of 0.90 [51].

## Investigations in patients with hyperthermia or pyrexia

There is a relative lack of data pertaining to the approach to determining the aetiology of pyrexia. In a multinational survey of temperature management practices in 139 general medical–surgical ICUs, 59 % of respondents indicated that new pyrexia triggers a full septic work-up, mostly via specific physician order, not a standardized protocol [22]. In a study examining management practices among febrile critically ill adults without an acute neurological condition, Niven et al. [36] found that 89 % of the population had at least one culture sent to the laboratory for analysis within the first 48 hours of fever onset. The most common culture was a blood culture (73 %), followed by urine culture (62 %) and respiratory secretion culture (61 %). A chest radiograph was ordered in nearly all study participants (95 %) within 48 hours of fever onset [36].

Not surprisingly, given the paucity of studies describing the approach to determining the aetiology of pyrexia in patients admitted to ICUs, there is a similar lack of data describing the yield of such investigations in febrile patients. A series of three retrospective cohort studies in trauma patients admitted to one surgical/trauma ICU in the United States examined the relationship between the presence of fever and/or leukocytosis and: the ordering of urine [52], blood [53], and respiratory secretion [54] cultures; or the occurrence of urinary tract, bloodstream, and lower respiratory tract infections, respectively. For each fluid cultured, the results were generally the same; namely, a strong relationship between the presence of pyrexia and the ordering of urine [52], blood [53], and respiratory secretion [54] cultures, but no significant relationship between the presence of pyrexia and positive culture from the urine, blood, or lower respiratory tract. In a meta-analysis, Coburn et al. [55] examined clinical and laboratory features predictive of bacteremia in immunocompetent adults. The majority of the 35 included studies did not include critically ill patients; however, according to their meta-analysis, irrespective of the severity of pyrexia, elevated body temperature was not independently associated with the presence of bacteremia [55]. In addition, the absence of pyrexia was not sufficient to rule out bacteremia (temperature ≥ 38.3 °C, negative likelihood ratio (LR) 0.80, 95 % CI 0.61–1.0). The presence of shaking chills or rigors was modestly predictive of bacteremia (positive LR 4.7, 95 % CI 3.0–7.2), whereas the absence of the systemic inflammatory response syndrome (SIRS) was the strongest predictor of negative blood cultures (negative LR 0.09, 95 % CI 0.03–0.26).

Guidelines recommend a clinically driven, cost-conscious approach, rather than a protocolized, dogmatic approach, to obtaining cultures and imaging studies in critically ill patients with pyrexia [19]. Based on existing literature that suggests a poor association between pyrexia and the likelihood of a positive culture, yet a high likelihood that pyrexia heralds the presence of an infection, most infections are likely diagnosed based on clinical and radiographic findings. Therefore, we suggest for immunocompetent patients with an elevated body temperature that investigations should be guided by the clinical probability of the aetiologies outlined in Fig. 1. Blood cultures should be obtained in any patient with rigors, and may be avoided in febrile patients without concomitant SIRS.

## Conclusions

Whether due to pyrexia or a hyperthermia syndrome, elevated body temperature is commonly encountered in patients admitted to adult ICUs. Although estimates of the incidence of infectious and noninfectious aetiologies are derived from studies with small sample size and inconsistent reporting of noninfectious aetiologies, current literature suggests that pyrexia is most frequently the sign of an infection. Among patients admitted to the ICU following surgery and/or an acute neurological condition, early fever may indicate a noninfectious process. Pyrexia commonly triggers a full septic work-up, but on its own is a poor predictor of culture-positivity. In order to improve culturing practices, and better guide the diagnostic approach to critically ill patients with pyrexia, additional research is required to provide more robust estimates of the incidence of infectious and noninfectious aetiologies, and their relationship to other clinical features. In the meantime, we suggest that investigations in patients with an elevated body temperature should be guided by the clinical probability of the aetiologies outlined in our proposed diagnostic approach.

## Abbreviations

LR, likelihood ratio; OR, odds ratio; SIRS, systemic inflammatory response syndrome
